# The influence of the carrier molecule on amoxicillin recognition by specific IgE in patients with immediate hypersensitivity reactions to betalactams

**DOI:** 10.1038/srep35113

**Published:** 2016-10-12

**Authors:** Adriana Ariza, Cristobalina Mayorga, María Salas, Inmaculada Doña, Ángela Martín-Serrano, Ezequiel Pérez-Inestrosa, Dolores Pérez-Sala, Antonio E. Guzmán, María I. Montañez, María J. Torres

**Affiliations:** 1 Research Laboratory, IBIMA–Regional University Hospital of Malaga–UMA, Málaga, Spain; 2 Allergy Unit, IBIMA–Regional University Hospital of Malaga–UMA, Málaga, Spain; 3 Andalusian Center for Nanomedicine and Biotechnology - BIONAND, Málaga, Spain; 4 Department of Organic Chemistry, University of Málaga, IBIMA, Málaga, Spain; 5 Centro de Investigaciones Biológicas, CSIC, Madrid, Spain; 6 Pharmacy Unit, Regional University Hospital of Malaga, Málaga, Spain

## Abstract

The optimal recognition of penicillin determinants, including amoxicillin (AX), by specific IgE antibodies is widely believed to require covalent binding to a carrier molecule. The nature of the carrier and its contribution to the antigenic determinant is not well known. Here we aimed to evaluate the specific-IgE recognition of different AX-derived structures. We studied patients with immediate hypersensitivity reactions to AX, classified as selective or cross-reactors to penicillins. Competitive immunoassays were performed using AX itself, amoxicilloic acid, AX bound to butylamine (AXO-BA) or to human serum albumin (AXO-HSA) in the fluid phase, as inhibitors, and amoxicilloyl-poli-L-lysine (AXO-PLL) in the solid-phase. Two distinct patterns of AX recognition by IgE were found: Group A showed a higher recognition of AX itself and AX-modified components of low molecular weights, whilst Group B showed similar recognition of both unconjugated and conjugated AX. Amoxicilloic acid was poorly recognized in both groups, which reinforces the need for AX conjugation to a carrier for optimal recognition. Remarkably, IgE recognition in Group A (selective responders to AX) is influenced by the mode of binding and/or the nature of the carrier; whereas IgE in Group B (cross-responders to penicillins) recognizes AX independently of the nature of the carrier.

Allergy to antibiotics is a major health problem in Europe, with betalactams (BLs) the most frequent culprits^1^,^2^. Approximately 10% of the population reports this allergy^3^, however, less than 24% of these cases can be confirmed^1^,^4^, perhaps due to the low sensitivity of the available diagnostic methods where the proper drug derivative involved in the reaction is not probably included. Moreover, although BLs specific IgE (sIgE) determination is valuable, its predictive value is not very high, therefore it should be performed in combination with skin test or drug provocation test (DPT) to get an accurate diagnosis^5^.

All BLs used in clinical practice can induce allergy, but amoxicillin (AX), with or without clavulanic acid, is the most common elicitor^2^,^6^. The chemical structure of AX is composed of a ß-lactam ring fused to a thiazolidine ring and a side chain (2-Amino-2-(4-hydroxyphenyl)acetamido) bound to carbon 6 of the penicillin (Fig. 1). Similarities and differences in the chemical structure of AX compared to other BLs help explain why some patients develop allergy only towards AX and tolerate other BLs (selective reactors)^7^,^8^,^9^, whilst others react to several BLs (cross-reactors)^2^,^8^,^10^,^11^,^12^,^13^.

AX is a low molecular weight molecule that, according to the hapten hypothesis, does not induce an immune response unless covalently bound to a carrier, usually a protein^14^, in order to give rise to sufficiently large size^15^,^16^. This process occurs through the opening of the ß-lactam ring by the amino groups of protein lysine residues^17^, forming the amoxicilloyl (AXO) antigenic determinant (Fig. 1)^17^,^18^. In the degradation pathway of AX, other structures such as amoxicilloic acid (result from β-lactam ring hydrolysis) and diketopiperazine (resulting from intramolecular acylation by the amino group of AX side chain) can be formed^19^. These two structures do not have the ability to bind covalently to proteins and correspondingly, as demonstrated in skin test and basophil activation test, cannot be recognized by sIgE from allergic patients^20^. Moreover, monoclonal antibodies to AX have shown that, besides the side chain, part of the carrier molecule is needed to achieve optimal recognition^21^. Additionally, other studies indicate that test sensitivity depends on the carrier molecule but that also the density and distribution of the AXO can have an important role^22^,^23^,^24^. Taken together, these studies suggest that the immune response to AX is determined not only by its chemical structure but also by the nature of the carrier molecules^14^.

Traditionally, human serum albumin (HSA) has been considered the main AX target because is the most abundant serum protein and possesses a very high ligand-binding capacity^25^. In addition, other serum proteins such as transferrin and immunoglobulins^26^ and also intracellular proteins from monocytes, B-lymphoma cells, and macrophages cell lines have been reported as AX target carriers in *in vitro* studies^27^. From an immunological point of view, the relevant carrier proteins have not been fully identified. The *in vivo* haptenation process is complex^28^ and could be one of the main limitations for detecting drug-protein adducts generated *in vivo* after drug administration. However, mass spectrometry (MS) techniques have recently allowed the characterization of HSA modified by AX in serum from subjects under oral AX treatment^26^,^29^, having previously been performed for other BLs (benzylpenicillin (BP), flucloxacillin and piperacillin)^30^,^31^,^32^,^33^,^34^.

The characterization of the AX determinants, the endogenous candidate carrier protein and the IgE recognition of the conjugate is necessary to understand the mechanisms of allergy as well as to implement diagnostic tests^35^,^36^,^37^,^38^,^39^. Therefore in this work we have studied sIgE recognition in AX allergic patients for four different structures derived from AX. Two of the structures are not bound to a carrier: AX itself and amoxicilloic acid, with only the former retaining the capacity to bind to proteins forming the AXO group. The other two structures consist of AX bound to two different carrier molecules, butylamine (BA) or HSA: both have the AXO groups, however the former is a monomer presenting a single AXO antigenic determinant, whilst the latter is a macromolecule with several AXO groups.

## Experimental Design

In order to perform this study, a group of patients with immediate allergic reaction to AX were selected according to the European Network for Drug Allergy (ENDA) guidelines and further classified as selective or cross-reactors to BLs.

With the aim of evaluating which structures derived from AX were recognized by IgE from allergic patients to this drug, radio-allergo-sorbent-test (RAST) inhibition assay was performed using AX and three derivatives (amoxicilloic acid, AXO-BA and AXO-HSA) at different concentrations as inhibitors. For further comparisons between groups of patients, the 1 mM concentration of AX derivatives was selected, since it was the optimal concentration that produced a differential behaviour in IgE recognition. This was carried out using the Mann–Whitney test for non-related samples and Wilcoxon test for related samples.

Given that AX can react during the RAST inhibition assay, through either degradation (to amoxicilloic acid or diketopiperazine) or conjugation to the proteins from patient sera, the formation of AX-derived molecules was studied. First, AX-protein conjugates were analysed by SDS-PAGE (sodium dodecyl sulphate polyacrylamide gel electrophoresis) and Western blot using an anti-AX monoclonal antibody and, second, the content of low molecular weight compounds was studied by spectroscopic techniques and liquid chromatography. Finally, in order to study their ability to be recognized by IgE and if there are differences depending on the groups, different molecular weight fractions of these AX-modified sera were employed as inhibitors in RAST inhibition assays.

## Results

### Immunological recognition of AX-derived structures

We analysed the relevance and influence of carrier molecules on the recognition of AX by sIgE in 21 allergic patients with a confirmed diagnosis of an immediate allergic reaction to AX. All cases were diagnosed by skin test positive to BP or AX determinants and RAST positive to amoxicilloyl-poly-L-lysine (AXO-PLL). This group of patients was selected from our databank (2000–2015) that includes 751 patients allergic to BLs. All patients were re-evaluated before inclusion. The demographic, clinical, and serologic characteristics of the patients are shown in Table 1. Twelve were women (57%) and the median age was 43 (interquartile range (IR): 36–51) years old. The clinical entities observed were anaphylactic shock for 3 (14.2%), anaphylaxis for 12 (57.1%) and urticaria for 6 cases (28.5%). The median time interval between the reaction and the study was 5 (IR: 2–8.5) months.

According to the IgE recognition to BLs, patients showed two well-differentiated patterns: patients with selective allergic reactions to AX (Group A), and patients who had a cross-reaction (Group B). Patients with selective reactions to AX are defined as those with negative skin test to BP determinants but positive to AX, with good tolerance to BP and penicillin V, and sIgE antibodies to AX but no detection of sIgE to BP; patients with cross-reactive reactions have positive skin test to BP determinants and/or AX and present sIgE to BP and AX (Table 1). We found that there were no differences in gender, age (Group A, median: 44 years, IR: 39–47.75 years; Group B, median: 40 years, IR: 29.5–58.5 years) and severity of the reaction between both groups. We did find significant differences in the time interval between reaction and study (p = 0.003) (Group A, median: 8.5 months, IR: 5.5–14 months; Group B, median: 2 months, IR: 1–5 months).

**Figure 1:**
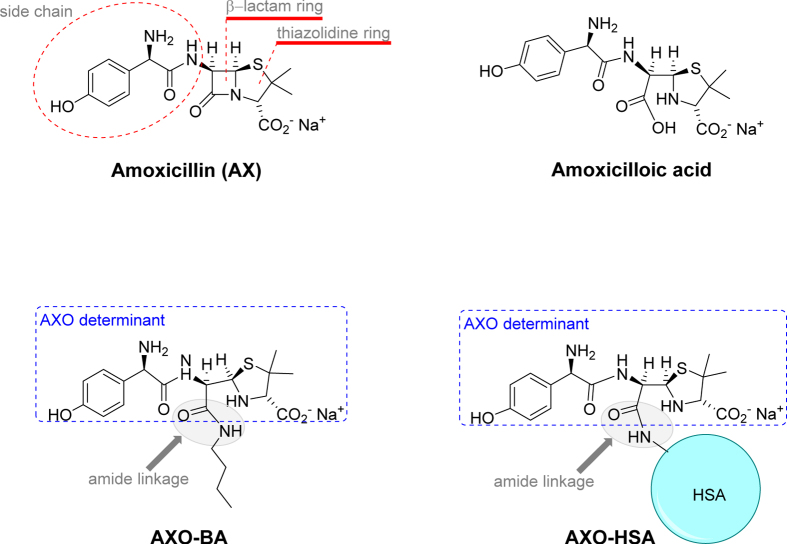
Chemical structure of the AX-derived molecules: amoxicillin (AX), amoxicilloic acid, amoxicilloyl-butylamine (AXO-BA) and amoxicilloyl-human serum albumin (AXO-HSA) used as inhibitors in the RAST inhibition assay.

**Figure 2:**
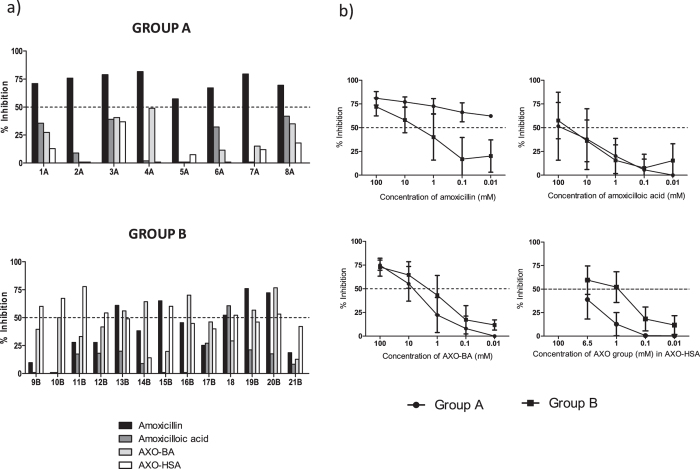
RAST inhibition assay results. (**a**) RAST inhibition assay performed with individual sera from AX allergic patients, with selective response to AX, Group A (Top) and, with cross-reactivity to BP and AX, Group B (Bottom). Solid-phase: cellulose discs modified with AXO-PLL. Inhibitors: AX (1 mM), amoxicilloic acid (1 mM), AXO-BA (1 mM) and AXO-HSA (1 mM AXO groups bound to HSA). Results are expressed as percentage of inhibition. (**b**) RAST inhibition assay performed with serum samples of AX allergic patients from Group A (filled circles) and Group B (filled squares). Solid-phase: cellulose discs modified with AXO-PLL. Inhibitors: AX (100–0.1 mM), amoxicilloic acid (100–0.1 mM), AXO-BA (100–0.1 mM) and AXO-HSA (6.5–0.01 mM AXO groups bound on HSA). Results are expressed as mean ± SD of percentage of inhibition for each concentration.

The *in vitro* analyses of the IgE recognition were performed by RAST inhibition assay using sera with RAST values to AXO-poly-L-lysine (AXO-PLL) higher than 7%. We used as solid-phase AXO-PLL cellulose discs, and as fluid phase different inhibitors: AX itself, amoxicilloic acid, AXO-BA and AXO-HSA (Fig. 1). We observed two well-defined patterns considering *in vitro* IgE recognition (inhibition >50%) to these structures that was well related with the clinical response in both Group A and Group B (Fig. 2a). In Group A (N = 8), all patients presented a relevant IgE recognition of AX itself, with no IgE recognition of the rest of the inhibitors (Fig. 2a, top). In Group B (N = 13), 8 cases had relevant IgE recognition of AXO-HSA with no cases having IgE recognition only of AX itself (Fig. 2a, bottom). However the pattern of IgE recognition was more heterogeneous than in Group A, with 4 cases having more than 50% of inhibition only of AXO-HSA, 3 of AX, AXO-BA and AXO-HSA, 2 of AXO-BA, 1 of AX and AXO-BA, 1 of AX, amoxicilloic acid and AXO-HSA and 2 with no relevant recognition.

We compared the mean ± SD percentage of inhibition results at decreasing inhibitor concentrations (100–0.01 mM for AX, amoxicilloic acid and AXO-BA and 6.5–0.01 mM of AXO groups in AXO-HSA adducts) between groups (Fig. 2b). At the maximum concentration of most of inhibitors (100 mM), low differences were found between groups; however, at decreasing concentrations different patterns clearly appeared between groups A and B, particularly for AX itself. In Group A, only a slight decrease in the percentage of inhibition (from 80.93% to 62.30%) was found even at the lowest AX concentration (0.01 mM). However, in Group B, we found a strong decrease on percentage of inhibition to 40.02% at 1 mM and 16.90% at 0.1 mM of AX inhibitor. Considering amoxicilloic acid, no differences were found between both groups at any concentration without relevant IgE recognition. Finally, we observed similar results for AX conjugated to BA or HSA, with both Group A and Group B having parallel curves although IgE recognition was always higher in Group B. Interestingly, no recognition of AXO-HSA was observed in Group A, for any of the concentrations used.

In Group B, the half maximal inhibitory concentration (IC50) for AX is in the range between 10–1 mM whereas in Group A more than 100 fold lower concentration is needed (Fig. 2b). No differences were found in the IC50 for amoxicilloic acid and AXO-BA. In the case of AXO-HSA, IC50 is around 1 mM in Group B and at least 10 fold greater concentration is needed in Group A.

**Figure 3:**
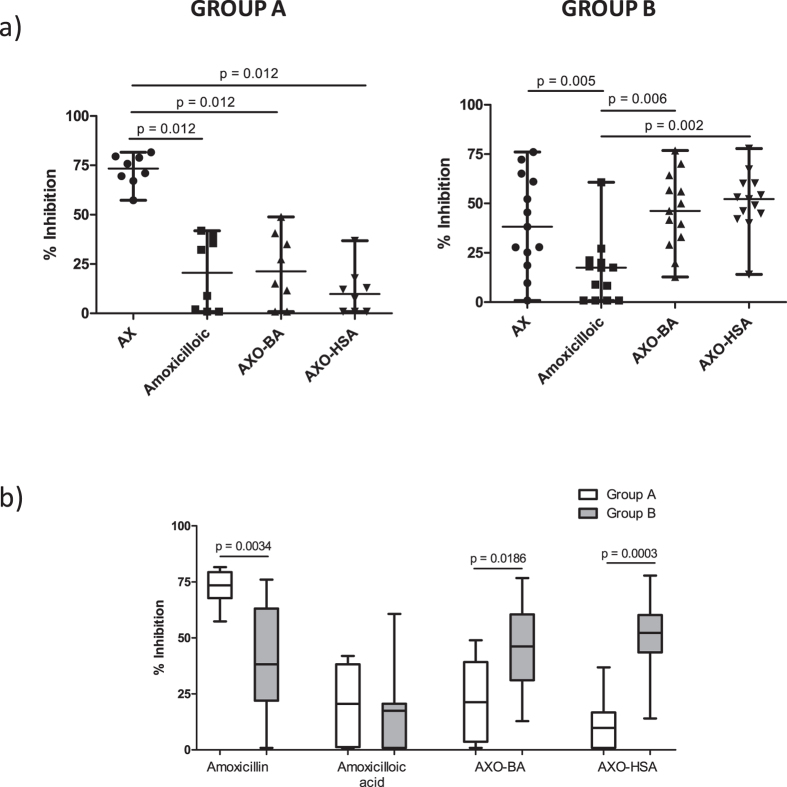
Comparisons of RAST inhibition results. (**a**) Comparison of RAST inhibition values for each inhibitor at a concentration of 1 mM of AX, amoxicilloic acid and AXO-BA and 1 mM of AXO determinants in AXO-HSA in sera for Group A (left) and Group B (right) patients, performed using non-parametric analysis for related samples, Wilcoxon test. Significant differences were considered when p < 0.05. (**b**) Comparison of RAST inhibition values for each inhibitor at a concentration of 1 mM of AX, amoxicilloic acid and AXO-BA and 1 mM of AXO determinants in AXO-HSA between groups A and B performed using non-parametric analysis for non-related samples, Mann-Whitney test. Significant differences were considered when p < 0.05.

Analysing the differences between inhibitors in each group in detail (Fig. 3a), we observed that in Group A, there was significantly higher IgE recognition of AX itself (median:73.40%; IR:67.70–79.35%) compared to amoxicilloic acid (median: 20.55%; IR: 1.175–38.20%; p = 0.012), AXO-BA (median: 21.25%; IR: 3.575–39.20%; p = 0.012) and AXO-HSA (median: 9.750%; IR: 0.9–16.65%; p = 0.012). No differences were found between the two AXO conjugates and amoxicilloic acid in this group. However, in Group B, the recognition of amoxicilloic acid was significantly lower (median: 17.44%; IR: 0.9–20.59%) than that of AX itself (median: 38.23%; IR: 21.91–63.05%; p = 0.005), AXO-BA (median: 46.20%; IR: 31.07–60.48%; p = 0.006) and AXO-HSA (median: 52.20%; IR: 43.50–60.15%; p = 0.002) (Fig. 3a). In this group, no differences between the two AXO conjugates and AX itself were found.

Analysing differences for each inhibitor between both groups (Fig. 3b), we observed a significantly higher recognition in Group A compared to Group B of AX (p = 0.0034) and higher recognition in Group B compared to Group A of AXO-BA (p = 0.0186) and AXO-HSA (p = 0.0003). No significant differences between groups were observed for amoxicilloic acid, which was the least well recognized structure.

### Study of AX reactivity in sera during *in vitro* immunoassay

**Figure 4:**
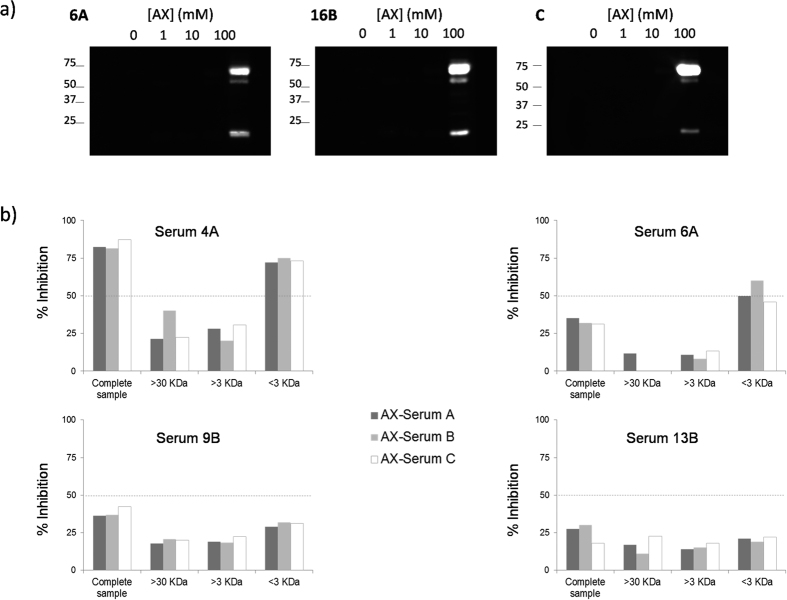
Detection of serum proteins modified with AX and study of their IgE recognition. (**a**) Immunological detection by Western blot of serum proteins modified with AX during inhibition RAST procedure. Three representative cases from a selective allergic patient to AX (6A), a cross-reactive patient to penicillins (16B) and a healthy control C are shown. (**b**) RAST inhibition assays of different serum samples (4A, 6A, 9B and 13B) from AX allergic patients. Solid-phase: cellulose discs modified with AXO-PLL. Inhibitors: Different fractions (complete sample (CS), fraction with components of molecular weight higher than 30 KDa (FC > 30), fraction with components of molecular weight higher than 3 KDa (FC > 3), fraction with components of molecular weight lower than 3 KDa (FC < 3)) of three AX-modified-sera (from group A, B and tolerant control) at protein concentration 1 mg/mL for CS, FC > 30 and FC > 3, and at the same dilution as FC > 3 for FC < 3). Results are expressed as percentage of inhibition.

As AX itself was significantly better recognized by IgE in Group A than in Group B, and as this structure is the only inhibitor that retains its ability to conjugate to proteins, we considered it important to analyse whether AX suffers conjugation to the proteins from patients sera during the RAST inhibition assay and if there were differences depending on the groups. To verify this, we detected the AX-protein adducts formed in different sera by SDS-PAGE and Western blot using an anti-AX monoclonal antibody. Figure 4a shows the AX-protein adducts detected in serum from patients in Group A, Group B and tolerant controls. Results indicate that the same band pattern corresponding to the major AX-serum targets, namely, HSA and the light and heavy chains of immunoglobulins, appeared in all cases. Regarding the components of low molecular weight fractions (FC < 3 KDa) the nuclear magnetic resonance (NMR) and high-performance-liquid-chromatography (HPLC) analyses confirmed the presence of AX itself, amoxicilloic acid and diketopiperazine whereas the matrix-assisted-laser-desorption/ionization time-of-flight (MALDI-TOF) MS shows the presence of small AX-modified molecules (Figure 4-Supplementary).

### Immunological recognition of AX-modified molecules formed in sera during immunoassay

The IgE recognition of the different modified sera fractions was analysed by RAST inhibition using sera from groups A and B (Fig. 4b). The results indicated a higher recognition of the AX derivatives contained in the FC < 3 by IgE from Group A and no differential recognition of any of the fractions by IgE from Group B.

## Discussion

The formation of hapten-protein conjugates is a key process in the pathogenesis of AX allergic reactions, and although the chemical structure of the antigenic determinant (AXO) is well defined^17^,^18^, the nature of the proteins involved and their relevance is not completely known^40^,^41^,^42^. In order to understand how the carrier molecule(s) can influence the recognition by IgE in patients allergic to AX we have analysed several AX-derived structures: AX itself, amoxicilloic acid and AXO (AXO-BA and AXO-HSA) by RAST inhibition assay. Two main recognition patterns were found that interestingly were associated with the two types of clinical reactivity to AX: AX selective allergic patients better recognizing AX itself (Group A) and cross-reactive patients to BP and AX recognizing AX itself and AX previously conjugated to carrier molecules in a similar manner (Group B). Interestingly, there was a poor recognition of amoxicilloic acid in both groups, thus reinforcing the importance of the carrier molecule for optimal recognition. This low relevance of amoxicilloic acid in IgE recognition was already observed in a previous study indicating that the use of this molecule did not increase the positivity of skin test and basophil activation test^20^.

Analysing the clinical data and diagnosis of patients included in the study, there are some important aspects to detail. First, we found that the percentage of patients with anaphylaxis was 71%, which is similar to results obtained from groups from South Europe where this percentage ranged from 53.3% to 71%^11^,^43^. This indicates that although BL administration is mainly administered by oral route, anaphylaxis is not rare. Second, although false positive results have been demonstrated especially for penicillin V^5^, different publications have demonstrated a good correlation between the results of anti-AX and anti-BP IgE antibodies determined by radioimmunoassays and the response after DPT^11^,^43^,^44^,^45^. In our study, this agreement was higher between skin tests to AX and RAST to AXO-PLL because it was an inclusion criterion, although it was lower for BPO. In that sense, there were 9 patients with skin test negative to BP-OL and RAST positive to BPO-PLL.This could probably be due to the presence of false negative skin test results as previously published^45^.

The analysis of RAST inhibition results showed that these differences were apparent when using low concentrations of inhibitors. In that sense, we found that Group A had a consistent pattern of IgE recognition being AX itself the only relevant structure inducing IgE recognition even at the lowest concentration (0.01 mM).

The comparisons of IgE recognition between conjugates were done at the same AXO concentration (1 mM) with results demonstrating that the pattern of IgE recognition with AXO-HSA was equivalent to the one described with AXO-BA. Differences were only found between groups showing a higher recognition of both conjugates, AXO-BA and AXO-HSA, in Group B. These results confirm that IgE recognition has a different behaviour in both groups with AX being significantly better recognized in Group A and AXO-BA and AXO-HSA in Group B.

To interpret these results it should be taken into account that given its reactivity, AX can conjugate to sera proteins used in the RAST inhibition assay and therefore the resulting adducts are optimally recognized in Group A compared to the previously synthetized AXO-BA and AXO-HSA. On the other hand, amoxicilloic acid cannot covalently bind to serum proteins during the inhibition assay because the β-lactam ring is already open, and binding to proteins through other functional groups is unlikely in the assay conditions. This highlights the role of amide linkage in IgE recognition, since it is the only difference between amoxicilloic acid and AXO (Fig. 1) and, therefore, the importance of the haptenation process in the recognition of BL structures by the immune system.

Given the different patterns of recognition between groups, we investigated whether there is different conjugation behaviour among serum proteins of patients depending on their clinical reactivity. We analysed the production of adducts during the inhibition assay with the only structure retaining this capacity (AX itself). We observed that AX-modified-protein bands could be assigned to the major serum proteins, HSA, light and heavy chains of immunoglobulins, as previously reported^26^. Remarkably, no differences in the pattern of modification, at least for the major stable conjugates, were found between sera of patients from groups A and B or even from control subjects. Interestingly, although HSA was one of the proteins modified in the inhibition assay, when we used AXO-HSA as an inhibitor no patient from Group A optimally recognized this structure. This could be explained by the different conditions for adducts attainment, either *in vivo* or *in vitro* in terms of concentrations and pH, that could alter HSA conformation^46^,^47^,^48^. Therefore, the number^26^,^31^,^32^,^34^,^49^ and distribution^23^,^24^ of AXO molecules on HSA could also be different, and this could affect immunological recognition. The importance of the tridimensional conformation of the hapten in the adduct for IgE recognition has recently been emphasized using synthetic nano-carriers as a model^50^. Indeed, differences in the hapten spatial conformation were found, with the BPO group partially exposed to IgE recognition (only the thiazolidine ring) and with the side chain hidden, whereas the entire structure of the AXO group was exposed outside of the carrier molecule.

We looked in more detail at the recognition of AX by analysing specific binding of AX modified proteins contained in the complete sample or fractions of different molecular weight. IgE from Group A showed a specific recognition to AX-modified FC < 3 whereas no differences in recognition to different fractions were found in Group B. These results suggest that the nature of the carrier will influence the clinical response. Thus in selective responders, other molecules apart from the main serum proteins, HSA or immunoglobulins, especially those of low molecular weight, and maybe AX itself, seem to be more relevant for the optimal IgE recognition. In fact MS results seem to indicate that as-yet unidentified low molecular weight components from sera are modified with AX during the RAST inhibition procedure.

Summing-up, IgE recognition is influenced by the binding and nature of the carrier molecule in Group A, while IgE mainly recognizes the amoxicilloyl structure independently of the nature of the carrier in Group B. The sub-optimal IgE recognition of amoxicilloic acid reinforced the need for conjugation (through an amide linkage) to a carrier in Group B. These results lead to a better understanding of the haptenation process and will help improve diagnostic techniques.

## Methods

### Patients

Patients who had been diagnosed with an immediate allergic reaction to AX using the diagnostic procedure described in the ENDA^51^,^52^ were eligible for the study. In order to be included patients needed to have skin tests positive to BP determinants and/or AX and RAST positive to AXO-PLL higher than 7%, as this allowed performing the RAST inhibition assay.

Patients were classified as selective reactions to AX (Group A) when they had negative skin test to BP determinants, good tolerance in DPT to BP and penicillin V, and sIgE antibodies to AX but no detection of sIgE to BP. Patients were classified with cross-reactive reactions (Group B) when they had positive skin test to BP determinants and/or AX and present sIgE antibodies to BP and AX, and therefore DPT was not performed.

Blood samples collected from the included patients were processed following standard procedures by the Malaga Hospital-IBIMA Biobank. The study was conducted according to the Declaration of Helsinki principles and was approved by the Provincial Ethics Committee of Malaga. All subjects included in the study were informed orally and signed the corresponding informed consent.

### Skin testing

Skin prick tests (SPT) were performed as recommended and if negative were followed by intradermal tests (IDT)^51^,^52^. The maximum concentrations of the penicillin derivatives used were: benzylpenicilloyl-octa-L-lysine (BP-OL) 0.04 mg/mL (8.64 × 10^−5^ M of the benzylpenicilloyl (BPO) moiety); minor determinant (MD) 0.5 mg/mL (1.5 × 10^−3^ M of the sodium benzylpenilloate); AX 20 mg/mL (5 × 10^−2^ M). In the SPT, a wheal larger than 3 mm with a negative response to the control saline was considered positive. For IDT, the wheal area was marked at the start and again 20 minutes after testing. An increase in diameter greater than 3 mm was considered positive^53^.

### Drug provocation test (DPT)

This test was carried out in a single-blind procedure following ENDA recommendations^51^,^52^. DPT consists of the administration of increasing doses of the BL at regular time intervals (30–60 minutes) to achieve the therapeutic dose, followed by two days ambulatory administration. First intramuscular BP (Normon-Laboratories, Madrid, Spain) was administered at the clinical setting followed by two days of oral penicillin V (ERN, Barcelona, Spain) at home, followed one week later by oral AX (Glaxo-Smithkline, Madrid, Spain) at the clinical setting followed by two days of oral AX at home.

### SIgE antibodies determination

Serum sIgE from patients were quantified by using RAST with BP or AX conjugated to PLL (Sigma, St Louis, USA) bound to cyanogen bromide-activated cellulose discs^44^. RAST was performed incubating sera with the solid-phases for 3 h at room temperature. After washing, ^125^I-anti-IgEantibody (ALK-Abello, Madrid, Spain) was added for the detection and the radioactivity was measured in a gamma counter (Packard BioScience, Frankfurt, Germany). Results were calculated as a percentage of the maximum of the count per minute (cpm), as in Eq. (1), and considered positive if they were higher than 2.5% of label uptake, which was the mean ± 2 SD of the negative control group.


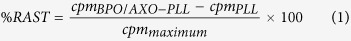


### Determination of IgE specificity and cross-reactivity

This was performed using the RAST inhibition assay^22^,^44^,^54^ with sera from patients with RAST values higher than 7%, using AXO-PLL solid-phase. As inhibitors (Fig. 1) we used AX, amoxicilloic acid, AXO-BA at 10-fold decreasing concentrations (100–0.01 mM) and AXO-HSA (at 6.5, 1, 0.1 and 0.01 mM of AXO determinants in AXO-HSA) in PBS that were incubated with serum for 3 h, at room temperature. Then, these fluid phases (inhibitors plus sera) were added to the solid-phase, AXO-PLL cellulose discs, and the RAST method was followed as described above. Results were expressed as percentage of inhibition calculated according to the Eq. (2):


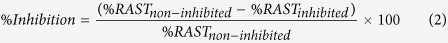


For further comparisons between groups of patients, we selected 1 mM of AX determinants. This was chosen because it was the optimal concentration that produced a differential behaviour in IgE recognition (Figure 1-Supplementary). Moreover, the IgE specificity to different derivatives was also determined by using the IC50.

### Preparation of inhibitor structures

Amoxicilloic acid and AXO-BA conjugates were prepared as described^20^,^44^ and characterized by NMR (Figure 2-Supplementary).

AXO-HSA was prepared by incubation of HSA (10 mg/mL) with a freshly prepared solution of AX (100 mg/mL) in 50 mM Na_2_CO_3_/NaHCO_3_ pH 10.2, for 48 h at 37 °C. AX unbound to proteins was removed by dialysis with 3 kDa cut-off membranes for 72 h in Na_2_CO_3_/NaHCO_3_ pH 10.2 buffer that was replaced every 12 h. AXO-HSA adducts were characterized by MALDI-TOF MS (Figure 3-Supplementary), the increment of mass observed (4850 Da), compared to the HSA control sample, corresponds approximately to 13 units of AXO conjugated per HSA molecule.

### *In vitro* modification of serum proteins of subjects (patients and controls) by AX for their use as inhibitors

Sera were incubated with a freshly prepared solution of AX (at 100 mM in 50 mM Na_2_CO_3_/NaHCO_3_ pH 10.2) for 48 h at 37 °C. Four fractions were obtained from the modified sera: i) complete sample (CS); ii) fraction with components of molecular weight higher than 30 KDa (FC > 30), dialyzed against PBS with filter membrane (30 KDa); iii) fraction with components of molecular weight higher than 3 KDa (FC > 3), dialyzed against PBS with filter membrane (3 KDa); iv) fraction with components of molecular weight lower than 3 KDa (FC < 3). FC > 30 and FC > 3 contain the drug bound to serum proteins and FC < 3 mainly contains the free drug or its metabolites.

Before using these fractions as inhibitors in RAST inhibition, the CS, FC > 30 and FC > 3 were normalized to 1 mg/mL protein concentration. For the FC < 3, as its low proteins content, the same dilution as FC > 3 was used. This fraction was analysed by NMR, MALDI-TOF MS and HPLC (Figure 4-Supplementary).

### SDS-PAGE electrophoresis and Western blot

The formation of AX-serum proteins adducts during the RAST inhibition assay, as a potential source of carriers, was assessed by collecting the sera samples after 3 h of incubation with AX (1, 10 and 100 mM). These sera samples were analysed by SDS-PAGE in 12.5% polyacrylamide gels. Afterwards, proteins were transferred to BioTrace PVDF membranes (0.45 μm, Pall Corporation, Life Sciences) on a wet blotting system (Bio-Rad, Hercules, CA, USA). AX-modified-proteins were detected using a mouse anti-AX monoclonal antibody at 1:500 dilution followed by incubation with a rabbit anti-mouse-Ig HRP-conjugated (Dako, Glostrup, Denmark) at 1:2000 dilution and enhanced chemiluminiscence detection^26^,^55^.

### Statistical analysis

Quantitative variables without a normal distribution were expressed as median and IR and comparisons were carried out using the Mann–Whitney test for non-related samples and Wilcoxon test for related samples. P values ≤  0.05 were considered statistically significant.

## Additional Information

**How to cite this article**: Ariza, A. *et al*. The influence of the carrier molecule on amoxicillin recognition by specific IgE in patients with immediate hypersensitivity reactions to betalactams. *Sci. Rep.* **6**, 35113; doi: 10.1038/srep35113 (2016).

## Supplementary Material

Supplementary Information

## Figures and Tables

**Table 1:** Classification and clinical characteristics of patients diagnosed with an immediate allergic reaction to AX included in the study.

Pat	Gen	Age (years)	Reaction	Adre	Drug	Route	Int D-R	Int R-S	Skin test			DPT BP/PV	% RAST	
									BP-OL	MD	AX		BPO-PLL	AXO- PLL
1A	F	35	Anaphylaxis	+	AX	O	30	7	−	−	+	−	1,09	21,79
2A	M	45	Anaphylaxis	nk	AX-Clav	O	nk	19	−	−	+	−	1,68	29,68
3A	F	42	Urticaria	−	AX	O	45	8	−	−	+	−	0,93	21,01
4A	M	43	Anaphylaxis	−	AX-Clav	O	30	5	−	−	+	−	0,00	25,58
5A	F	51	Urticaria	−	AX-Clav	O	30	11	−	−	+	−	0,31	23,61
6A	M	47	Anaphylactic shock	+	AX-Clav	O	15	15	−	−	+	−	0,00	15,37
7A	M	38	Anaphylaxis	+	AX-Clav	O	35	3	−	−	+	−	0,00	16,42
8A	F	48	Urticaria	−	AX	O	45	9	−	−	+	−	2,56	33,72
9B	F	19	Anaphylaxis	nk	AX	O	20	5	−	−	+	nd	51,17	26,30
10B	F	33	Anaphylaxis	nk	AX-Clav	O	20	2	+	−	+	nd	16,35	19,75
11B	M	65	Urticaria	−	AX	P	nk	10	−	−	+	nd	14,74	14,31
12B	M	50	Anaphylactic shock	+	AX	O	10	2	−	−	+	nd	16,85	17,13
13B	F	40	Anaphylaxis	+	AX-Clav	O	20	1	+	−	−	nd	23,90	16,58
14B	F	37	Anaphylaxis	+	AX	O	25	4	+	+	+	nd	41,84	60,43
15B	M	26	Anaphylaxis	−	AX	O	30	1	−	−	+	nd	6,87	21,62
16B	F	40	Anaphylactic shock	+	AX	O	15	5	−	−	+	nd	14,60	11,50
17B	F	57	Urticaria	−	AX	O	30	6	+	+	+	nd	25,53	28,92
18B	F	61	Urticaria	−	AX-Clav	O	40	2	−	−	+	nd	39,96	42,82
19B	M	51	Anaphylaxis	nk	AX	O	15	1	−	−	+	nd	16,33	23,20
20B	M	19	Anaphylaxis	+	AX	O	20	3	−	−	+	nd	15,23	10,33
21B	F	60	Anaphylaxis	+	AX-Clav	O	15	1	−	−	+	nd	33,28	19,89

Pat: Patient; Gen: Gender; Adre: Adrenalin treatment administered (+: yes; −: no; nk: not known); AX: amoxicillin; Clav: clavulanic acid; O: Oral; P: Parenteral; F: female; M: male; Int D-R: Time interval between drug administration and symptoms (min); Int R-S: Time interval between reaction and study (months); BP-OL: benzylpenicilloyl-octa-L-lysine; MD: minor determinant; DPT-BP/PV: Drug provocation test benzylpenicillin and penicillin V; BPO-PLL: benzylpenicilloyl-poly-L-lysine; AXO-PLL: amoxicilloyl-poly-L-lysine.

## References

[b1] Doña I. . Drug hypersensitivity reactions: response patterns, drug involved, and temporal variations in a large series of patients. J. Investig. Allergol. Clin. Immunol. 22, 363–371 (2012). 23101312

[b2] Torres M. J. & Blanca M. The complex clinical picture of beta-lactam hypersensitivity: penicillins, cephalosporins, monobactams, carbapenems, and clavams. Med. Clin. North Am. 94, 805–820, xii (2010). 20609864 10.1016/j.mcna.2010.04.006

[b3] Solensky R. Penicillin allergy as a public health measure. J. Allergy Clin. Immunol. 133, 797–798 (2014). 24332220 10.1016/j.jaci.2013.10.032

[b4] Zambonino M. A. . Diagnostic evaluation of hypersensitivity reactions to beta-lactam antibiotics in a large population of children. Pediatr. Allergy Immunol. 25, 80–87 (2014). 24329898 10.1111/pai.12155

[b5] Decuyper I. I. . Quantification of specific IgE antibodies in immediate drug hypersensitivity: More shortcomings than potentials? Clin. Chim. Acta 460, 184–189 (2016). 27376983 10.1016/j.cca.2016.06.043

[b6] Torres M. J. . The role of IgE recognition in allergic reactions to amoxicillin and clavulanic acid. Clin. Exp. Allergy 46, 264–274 (2016). 26662186 10.1111/cea.12689

[b7] Blanca M. . Anaphylaxis to amoxycillin but good tolerance for benzyl penicillin. *In vivo* and *in vitro* studies of specific IgE antibodies. Allergy 43, 508–510 (1988). 3232762 10.1111/j.1398-9995.1988.tb01628.x

[b8] Blanca M. . Allergy to penicillin with good tolerance to other penicillins; study of the incidence in subjects allergic to beta-lactams. Clin. Exp. Allergy 20, 475–481 (1990). 2253079 10.1111/j.1365-2222.1990.tb03139.x

[b9] Blanca M. . Side-chain-specific reactions to betalactams: 14 years later. Clin. Exp. Allergy 32, 192–197 (2002). 11929481 10.1046/j.1365-2222.2002.01299.x

[b10] Silviu-Dan F., McPhillips S. & Warrington R. J. The frequency of skin test reactions to side-chain penicillin determinants. J. Allergy Clin. Immunol. 91, 694–701 (1993). 8454791 10.1016/0091-6749(93)90188-l

[b11] Fontaine C. . Relevance of the determination of serum-specific IgE antibodies in the diagnosis of immediate beta-lactam allergy. Allergy 62, 47–52 (2007). 17156341 10.1111/j.1398-9995.2006.01268.x

[b12] Montañez M. I., Ariza A., Mayorga C., Fernandez T. & Torres M. Cross-Reactivity in Betalactam Allergy: Alternative Treatments. Curr. Treat. Options Allergy 2, 141–154 (2015).

[b13] Blanca M. . Immediate allergic reactions to betalactams: facts and controversies. Curr. Opin. Allergy Clin. Immunol. 4, 261–266 (2004). 15238790 10.1097/01.all.0000136764.74065.15

[b14] Landsteiner K. & Jacobs J. Studies on the Sensitization of Animals with Simple Chemical Compounds. J. Exp. Med. 61, 643–656 (1935). 19870383 10.1084/jem.61.5.643 PMC2133244

[b15] Lopez S. . Nonimmediate reactions to betalactams. Curr. Opin. Allergy Clin. Immunol. 7, 310–316 (2007). 17620822 10.1097/ACI.0b013e3281e209fe

[b16] Guengerich F. P. Mechanisms of drug toxicity and relevance to pharmaceutical development. Drug Metab. Pharmacokinet. 26, 3–14 (2011). 20978361 10.2133/dmpk.dmpk-10-rv-062 PMC4707670

[b17] Levine B. B. & Ovary Z. Studies on the mechanism of the formation of the penicillin antigen. III. The N-(D-alpha-benzylpenicilloyl) group as an antigenic determinant responsible for hypersensitivity to penicillin G. J. Exp. Med. 114, 875–904 (1961). 14464604 10.1084/jem.114.6.875 PMC2180410

[b18] Batchelor F. R., Dewdney J. M. & D. G. Penicillin allergy: the formation of the penicilloyl determinant. Nature 206, 362–364 (1965). 5835701 10.1038/206362a0

[b19] Ariza A. . Hypersensitivity reactions to Betalactams: Relevance of the hapten-protein conjugates. J. Investig. Allergol. Clin. Immunol. 25, 12–25 (2015). 25898690

[b20] Torres M. J. . Role of minor determinants of amoxicillin in the diagnosis of immediate allergic reactions to amoxicillin. Allergy 65, 590–596 (2010). 19968633 10.1111/j.1398-9995.2009.02245.x

[b21] Mayorga C. . Epitope mapping of beta-lactam antibiotics with the use of monoclonal antibodies. Toxicology 97, 225–234 (1995). 7716788 10.1016/0300-483x(94)02983-2

[b22] Blanca M. . Determination of IgE antibodies to the benzyl penicilloyl determinant. A comparison between poly-L-lysine and human serum albumin as carriers. J. Immunol. Methods 153, 99–105 (1992). 1517607 10.1016/0022-1759(92)90311-g

[b23] Park B. K., Tingle M. D., Grabowski P. S., Coleman J. W. & Kitteringham N. R. Drug-protein conjugates–XI. Disposition and immunogenicity of dinitrofluorobenzene, a model compound for the investigation of drugs as haptens. Biochem. Pharmacol. 36, 591–599 (1987). 3827946 10.1016/0006-2952(87)90707-6

[b24] Lee D., Dewdney J. M. & Edwards R. G. The influence of hapten density on the assay of penicilloylated proteins in fluids. J. Immunol. Methods 84, 235–243 (1985). 4067316 10.1016/0022-1759(85)90430-2

[b25] Fasano M. . The extraordinary ligand binding properties of human serum albumin. IUBMB Life 57, 787–796 (2005). 16393781 10.1080/15216540500404093

[b26] Ariza A. . Protein haptenation by amoxicillin: High resolution mass spectrometry analysis and identification of target proteins in serum. J Proteomics 77, 504–520 (2012). 23041134 10.1016/j.jprot.2012.09.030

[b27] Ariza A. . Study of protein haptenation by amoxicillin through the use of a biotinylated antibiotic. Plos One 9, e90891; 90810.91371/journal.pone.0090891 (2014). 10.1371/journal.pone.0090891 PMC3940954 24595455

[b28] Ariza A., Montanez M. I. & Perez-Sala D. Proteomics in immunological reactions to drugs. Curr. Opin. Allergy Clin. Immunol. 11, 305–312 (2011). 21659862 10.1097/ACI.0b013e3283489ae5

[b29] Garzon D. . Mass spectrometric strategies for the identification and characterization of human serum albumin covalently adducted by amoxicillin: ex vivo studies. Chem. Res. Toxicol. 27, 1566–1574 (2014). 25088930 10.1021/tx500210e

[b30] Yvon M., Anglade P. & Wal J. M. Identification of the binding sites of benzyl penicilloyl, the allergenic metabolite of penicillin, on the serum albumin molecule. FEBS Lett. 263, 237–240 (1990). 2335227 10.1016/0014-5793(90)81382-x

[b31] Whitaker P. . Mass spectrometric characterization of circulating and functional antigens derived from piperacillin in patients with cystic fibrosis. J. Immunol. 187, 200–211 (2011). 21606251 10.4049/jimmunol.1100647 PMC3145118

[b32] Jenkins R. E. . Characterisation of flucloxacillin and 5-hydroxymethyl flucloxacillin haptenated HSA *in vitro* and *in vivo*. Proteomics Clin. Appl. 3, 720–729 (2009). 21136982 10.1002/prca.200800222

[b33] El-Ghaiesh S. . Characterization of the antigen specificity of T-cell clones from piperacillin-hypersensitive patients with cystic fibrosis. J. Pharmacol. Exp. Ther. 341, 597–610 (2012). 22371438 10.1124/jpet.111.190900 PMC3362878

[b34] Meng X. . Direct evidence for the formation of diastereoisomeric benzylpenicilloyl haptens from benzylpenicillin and benzylpenicillenic acid in patients. J. Pharmacol. Exp. Ther. 338, 841–849 (2011). 21680886 10.1124/jpet.111.183871 PMC3164351

[b35] Blanca M. . Clinical evaluation of Pharmacia CAP System RAST FEIA amoxicilloyl and benzylpenicilloyl in patients with penicillin allergy. Allergy 56, 862–870 (2001). 11551251 10.1034/j.1398-9995.2001.00995.x

[b36] Torres M. J. . The diagnostic interpretation of basophil activation test in immediate allergic reactions to betalactams. Clin. Exp. Allergy 34, 1768–1775 (2004). 15544603 10.1111/j.1365-2222.2004.02110.x

[b37] Rodriguez-Pena R. . Potential involvement of dendritic cells in delayed-type hypersensitivity reactions to beta-lactams. J. Allergy Clin. Immunol. 118, 949–956 (2006). 17030251 10.1016/j.jaci.2006.07.013

[b38] Luque I. . *In vitro* T-cell responses to beta-lactam drugs in immediate and nonimmediate allergic reactions. Allergy 56, 611–618 (2001). 11421918 10.1034/j.1398-9995.2001.000115.x

[b39] Sanz M. L. . Flow cytometric basophil activation test by detection of CD63 expression in patients with immediate-type reactions to betalactam antibiotics. Clin. Exp. Allergy 32, 277–286 (2002). 11929494 10.1046/j.1365-2222.2002.01305.x

[b40] Levine B. B. & Fellner M. J. Immune responses to penicillin in man and penicillin allergy. J. Clin. Invest. 44, 1067 (1965). 10.1172/JCI105494 PMC292875 5926634

[b41] Park B. K., Pirmohamed M. & Kitteringham N. R. Role of drug disposition in drug hypersensitivity: a chemical, molecular, and clinical perspective. Chem. Res. Toxicol. 11, 969–988 (1998). 9760271 10.1021/tx980058f

[b42] Pichler W. J. Drug-induced autoimmunity. Curr. Opin. Allergy Clin. Immunol. 3, 249–253 (2003). 12865767 10.1097/00130832-200308000-00003

[b43] Torres J. . Diagnostic evaluation of a large group of patients with immediate allergy to penicillins: the role of skin testing. Allergy 56, 850–856 (2001). 11551249 10.1034/j.1398-9995.2001.00089.x

[b44] Moreno F. . Studies of the specificities of IgE antibodies found in sera from subjects with allergic reactions to penicillins. Int. Arch. Allergy Immunol. 108, 74–81 (1995). 7544181 10.1159/000237121

[b45] Torres M. J., Mayorga C., Cornejo-Garcia J. A., Romano A. & Blanca M. IgE antibodies to penicillin in skin test negative patients. Allergy 57, 965 (2002). 10.1034/j.1398-9995.2002.23832_10.x 12269955

[b46] Peters T. All about albumin: biochemistry, genetics and medical applications. (Academic Press, 1996).

[b47] van der Vusse G. J. Albumin as fatty acid transporter. Drug Metab. Pharmacokinet. 24, 300–307 (2009). 19745557 10.2133/dmpk.24.300

[b48] Ascenzi P. & Fasano M. Allostery in a monomeric protein: the case of human serum albumin. Biophys. Chem. 148, 16–22 (2010). 20346571 10.1016/j.bpc.2010.03.001

[b49] Lafaye P. & Lapresle C. Fixation of penicilloyl groups to albumin and appearance of anti-penicilloyl antibodies in penicillin-treated patients. J. Clin. Invest. 82, 7–12 (1988). 3392217 10.1172/JCI113603 PMC303468

[b50] Montanez M. I. . Recognition of multiepitope dendrimeric antigens by human immunoglobulin E. Nanomedicine 11, 579–588 (2015). 25661921 10.1016/j.nano.2015.01.006

[b51] Torres M. J. . Diagnosis of immediate allergic reactions to beta-lactam antibiotics. Allergy 58, 961–972 (2003). 14510712 10.1034/j.1398-9995.2003.00280.x

[b52] Blanca M. . Update on the evaluation of hypersensitivity reactions to betalactams. Allergy 64, 183–193 (2009). 19133923 10.1111/j.1398-9995.2008.01916.x

[b53] Brockow K. . General considerations for skin test procedures in the diagnosis of drug hypersensitivity. Allergy 57, 45–51 (2002). 11991289

[b54] Batchelor F. R., Dewdney J. M., Weston R. D. & Wheeler A. W. The immunogenicity of cephalosporin derivatives and their cross-reaction with penicillin. Immunology 10, 21–33 (1966). 4160334 PMC1423621

[b55] Sánchez-Gómez F. J. . Amoxicillin haptenates intracellular proteins that can be transported in exosomes to target cells. Allergy, doi: 10.1111/all.12958. (2016). 27319758

