# Supplementary material for: The influence of the carrier molecule on amoxicillin recognition by specific IgE in patients with immediate hypersensitivity reactions to betalactams
Source: Sci Rep. 2016 Oct 12;6:35113. doi: 10.1038/srep35113 (PMC5059705; doi:10.1038/srep35113)
Supplement: Supplementary Information [file srep35113-s1.pdf]

## Supporting Information

# The influence of the carrier molecule on amoxicillin recognition by specific IgE in patients with immediate hypersensitivity reactions to betalactams

Ariza A[1], Mayorga C[1,2], Salas M[2], Doña I[2], Martín-Serrano A[1,3], Pérez-Inestrosa E[3,4], Pérez-Sala D[5], Guzmán AE[6], Montañez MI[1,3], Torres MJ[2].

[1]Research Laboratory, IBIMA – Regional University Hospital of Malaga – UMA, Málaga, Spain.
[2]Allergy Unit, IBIMA – Regional University Hospital of Malaga – UMA, Málaga, Spain.
[3]Andalusian Center for Nanomedicine and Biotechnology - BIONAND, Málaga, Spain.
[4]Department of Organic Chemistry, University of Málaga, IBIMA, Málaga, Spain.
[5]Centro de Investigaciones Biológicas, CSIC, Madrid, Spain
[6]Pharmacy Unit, Regional University Hospital of Malaga, Málaga, Spain

**Figure 1 Supplementary**. RAST inhibition results (expressed as the mean + SD of percentage of inhibition) for allergic patients to AX. Solid phase: cellulose discs modified with AXO-PLL. Inhibitors: AX (100 - 0.1 mM), amoxicilloic acid (100 - 0.1 mM), AXO-BA (100 - 0.1 mM -) and AXO-HSA (6.5 - 0.02 mM of AXO groups bound on HSA).

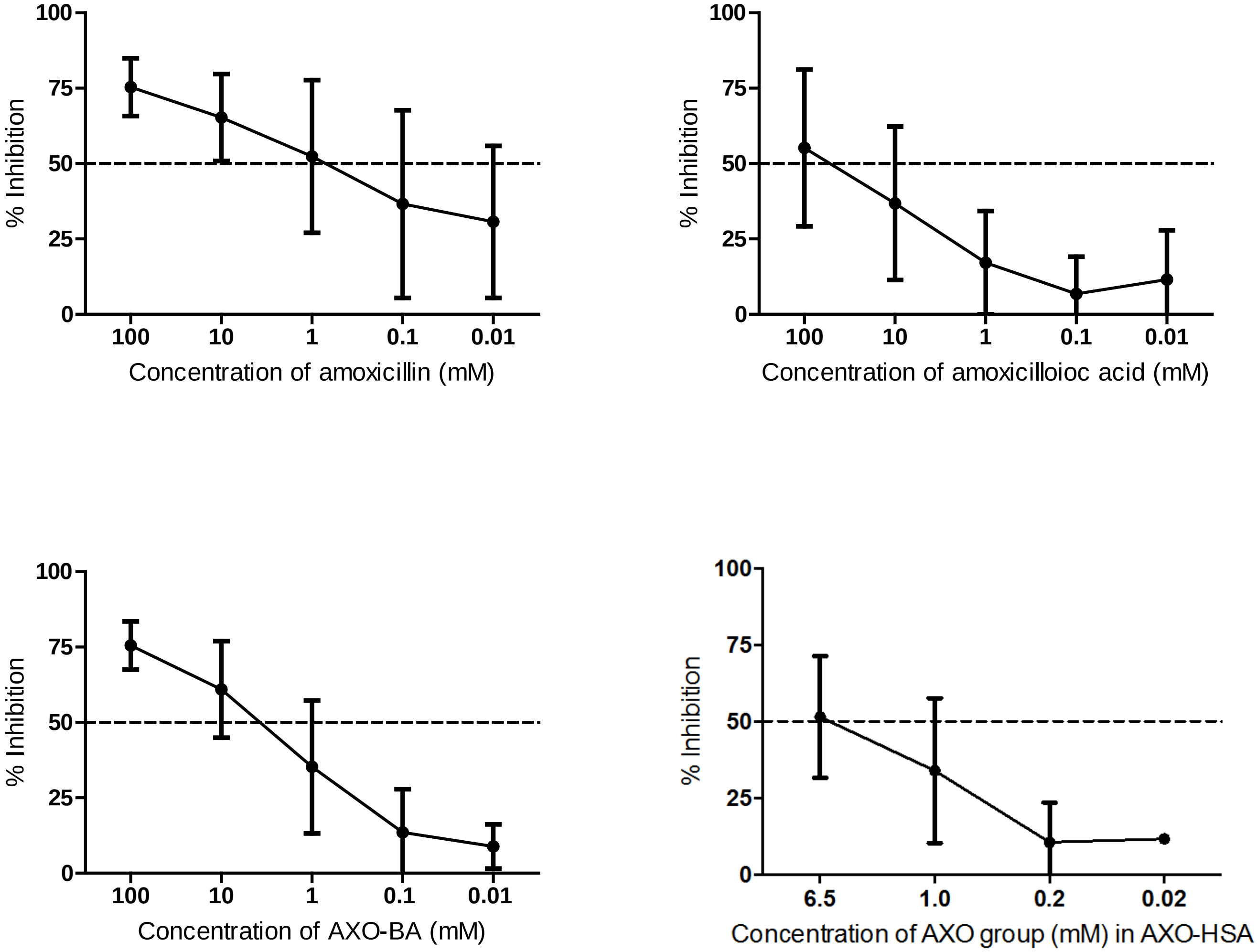

**Figure 2 Supplementary**. $^{1}$H-NMR spectra of inhibitors (amoxicilloic acid and HSA-AXO) in $D_2O$.

a) $^{1}$H-NMR spectra of amoxicilloic acid in $D_2O$

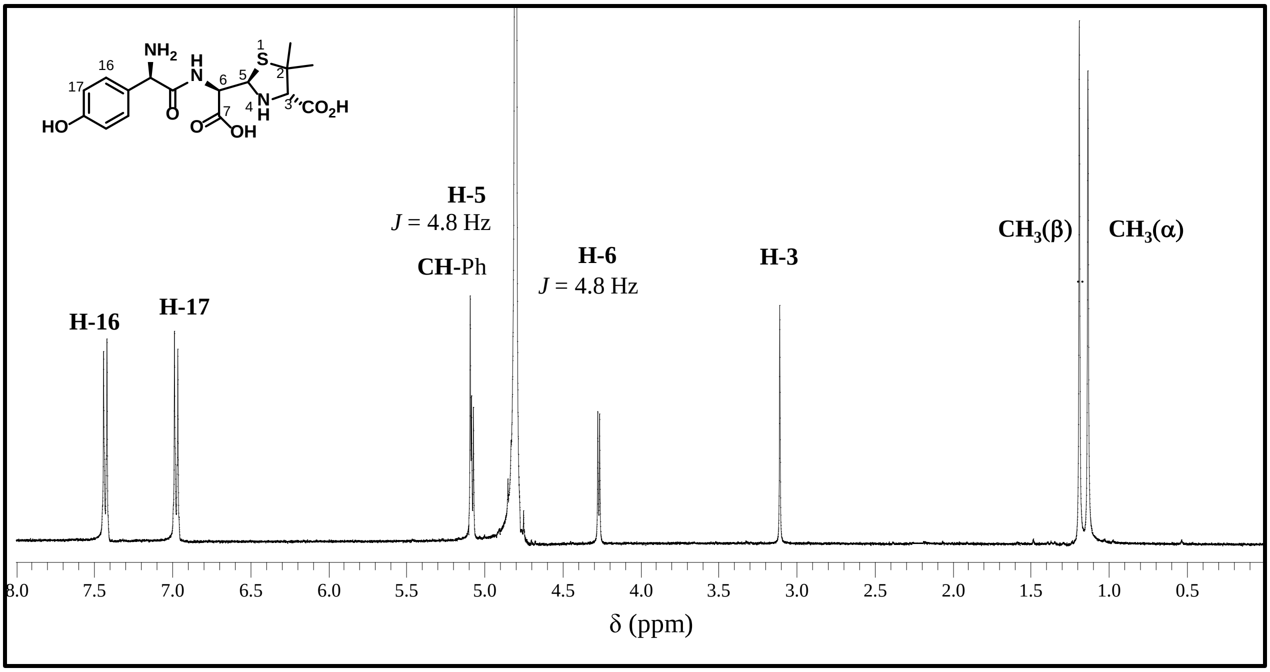


b) $^{1}$H-NMR spectra of AXO-BA in $D_2O$

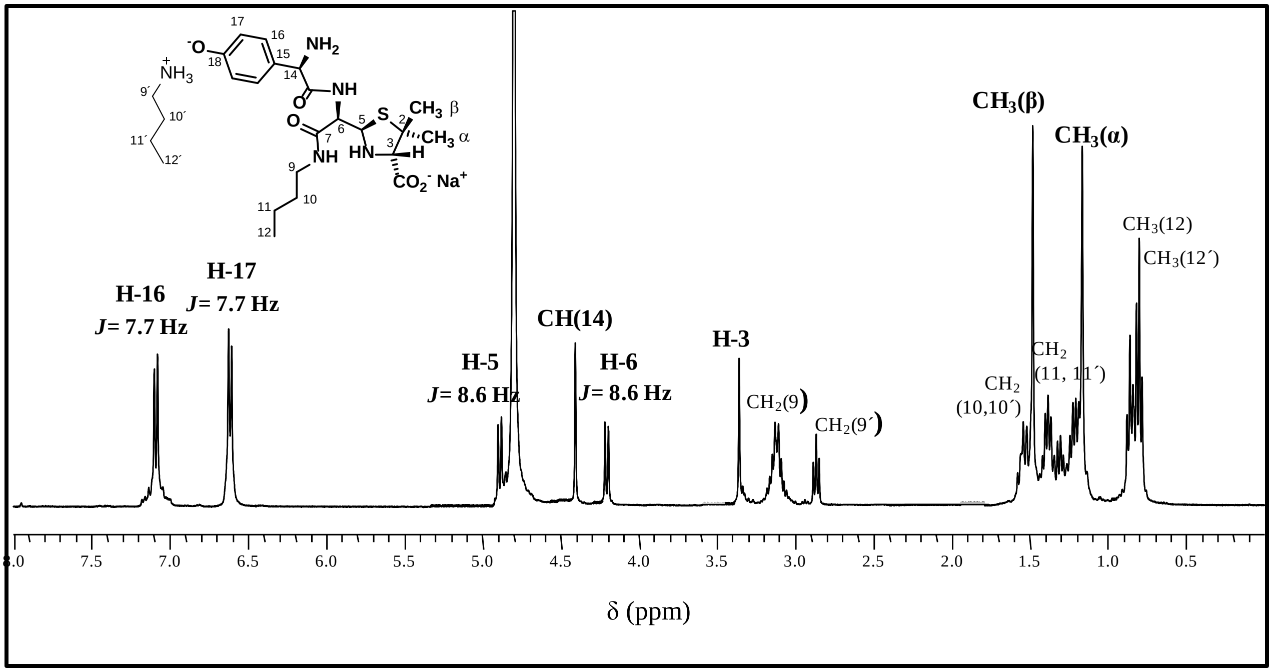

**Figure 3 Supplementary**. Overlapped MALDI-TOF MS spectra of HSA control sample (yellow) and HSA-AXO conjugate (violet).

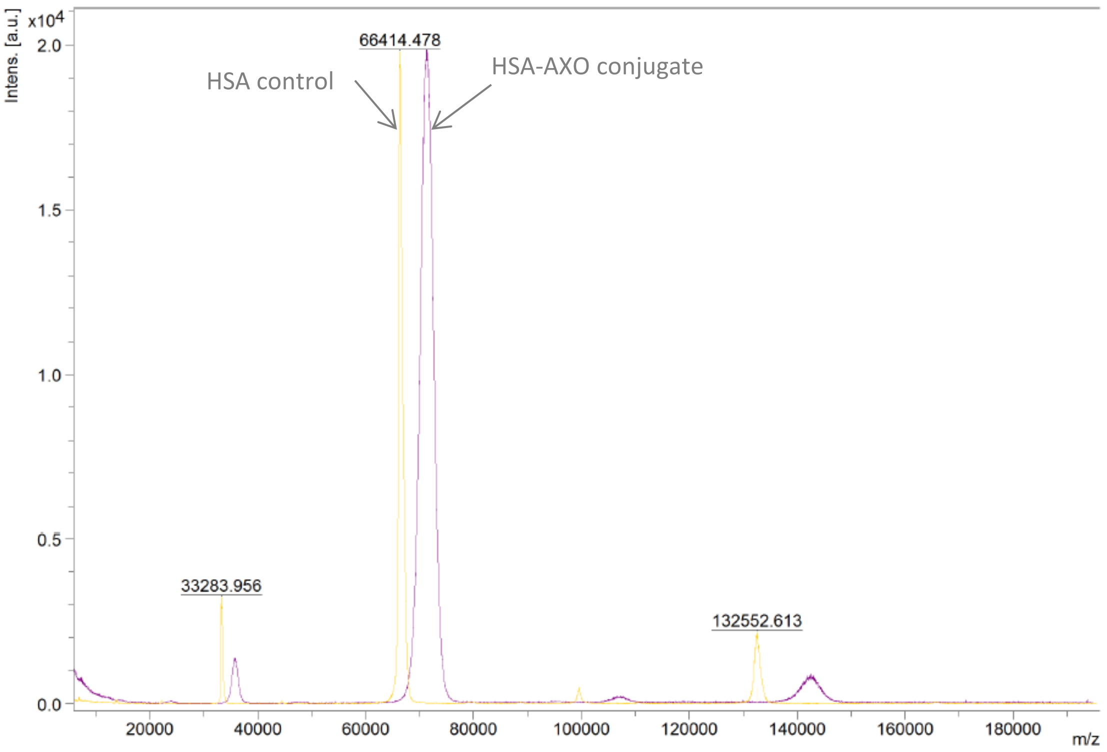


HSA control sample

| m/z | S/N | Quality Fac. | Res. | Intens. | Area |
|---|---|---|---|---|---|
| 33283.956 | 204 | | 5464 | 3238 | 33340 |
| 66414.478 | 1357 | | 10056 | 19803 | 365559 |
| 132552.613 | 146 | | 4170 | 2172 | 107493 |

HSA-AXO conjugate

| m/z | S/N | Quality Fac. | Res. | Intens. | Area |
|---|---|---|---|---|---|
| 35630.574 | | | | 1302 | |
| 71264.890 | 780 | | 16794 | 20075 | 151561 |
| 142781.519 | | | | 825 | |

**Figure 4a Supplementary**. $^1$H-NMR spectra of low molecular weight fraction <3 KDa of sera incubated with AX (lyophilized and solved in $D_2O$). Signals corresponding to AX, diketopiperazine (DKP) and amoxicilloic acid (AXO) and its enantiomer (C5) are identified.

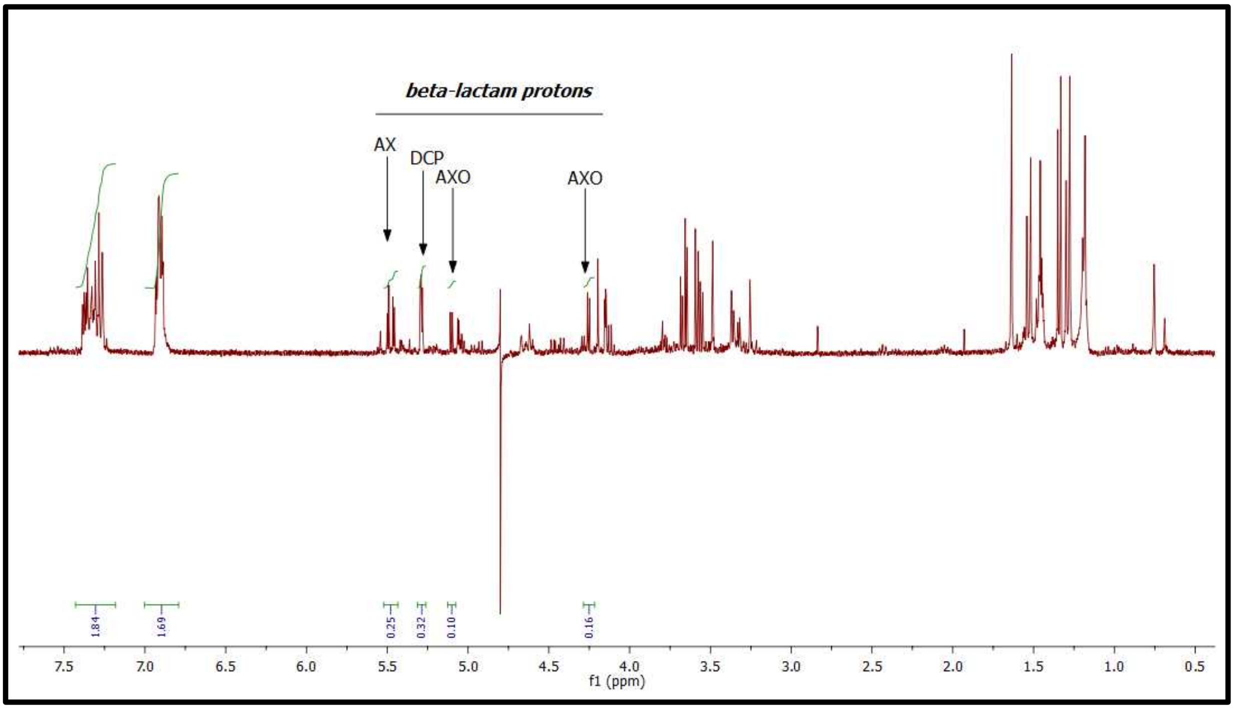


**Figure 4b Supplementary**. MALDI-TOF MS spectra of low molecular weight fraction <3 KDa of sera incubated with AX.

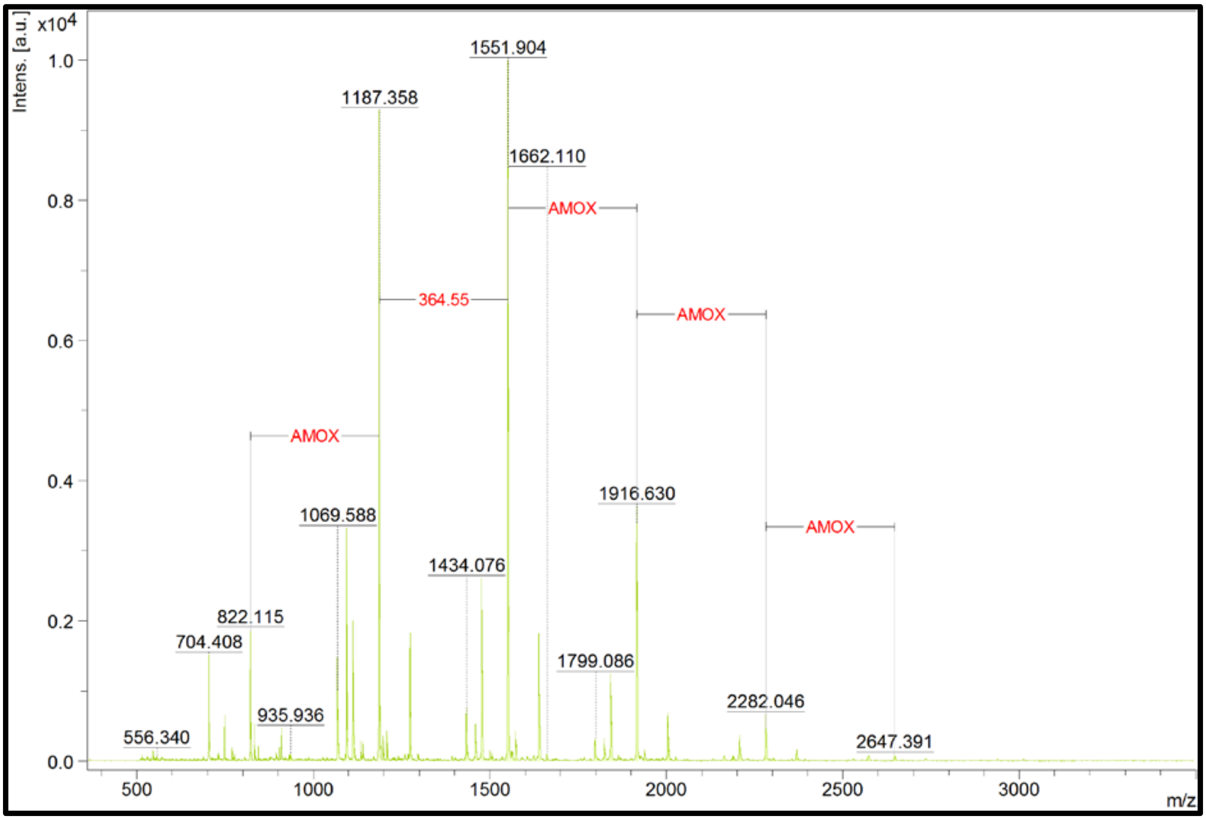

**Figure 4c Supplementary.** HPLC- H-ESI-MS of low molecular weight fraction <3 KDa of sera incubated with AX. AX, diketopiperazine (DKP) and two enantiomers of amoxicilloic acid were detected as indicated in the following table:

| Compounds | Amoxicillin (AX) | Amoxicilloic Acid (both enantiomers) | Diketopiperazine (DKP) |
|---|---|---|---|
| Quantification (μg/mL) | 81.5 | 484.34 | 524.3 |

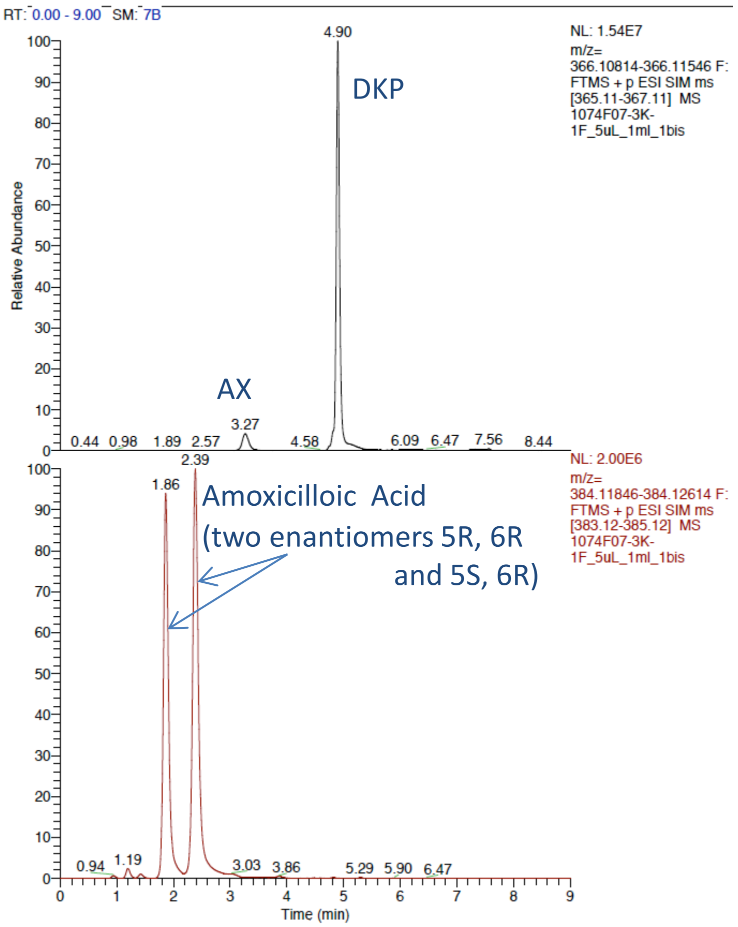


**Equipment:** Dionex Ultimate 3000 HPLC system and Orbitrap High Resolution Mass Spectrometer (Thermo Scientific). Ionization through electrospray by H-ESI-II
**Method:** Sample was lyophilized and solved, in miliQ water, for the analysis of AX and amoxicilloic acid, and in a mixture of miliQ water-methanol for the analysis of DKP.
Samples were injected into a Dionex Ultimate 3000 HPLC system equipped with a accucore RP-MS (50x2,1 mm ID, 2.6 μm) (Thermo Scientific), chromatographic column previously equilibrated with acetonitrile/amonic formiate pH:2.5 (2/98). Samples were eluted with isocratic flow 98/2 (acetonitrile/amoniac formiate pH:2.5) for 2 min, followed by gradient from 98/2 to 30/70 in 1 min, then isocratic mobil phase 30/70 (acetonitrile/amoniac formiate pH:2.5) for 2 min, and gradient from 30/70 to 98/2 (acetonitrile/amoniac formiate pH:2.5) for 3 min.
Analysis time: 9 min. Injection volume: 10 μL; Flow: 0.2 mL/min.
Detection was carried out with positive polarity, in selected ion monitoring (SIM mode), selecting 366.1118 ions (for AX and DKP) and 384.1223 (for amoxicilloic acid) and MS/MS mode for analytes confirmation.
